# Fatal Hemoptysis due to Chronic Cavitary Pulmonary Aspergillosis Complicated by Nontuberculous Mycobacterial Tuberculosis

**DOI:** 10.1155/2011/837146

**Published:** 2011-07-28

**Authors:** Ioannis Kokkonouzis, Ioannis Athanasopoulos, Nikolaos Doulgerakis, Grigorios Tsonis, Ioannis Lampaditis, Nikolaos Saridis, Vasilios Skoufaras

**Affiliations:** Department of Respiratory Medicine, Hellenic Air Force General Hospital, 11525 Athens, Greece

## Abstract

A 51-year-old man, with a history of severe COPD and bilateral pneumothorax, who was under treatment for pulmonary tuberculosis due to mycobacterium avium, was admitted due to high-grade fever, weight loss, cough, and production of purulent sputum, for almost one month without any special improvement despite adequate antibiotics treatment in outpatient setting. A CT scan revealed multiple consolidations, fibrosis, scaring, and cavitary lesions in both upper lobes with newly shadows which were fungus balls inside them. Aspergillus flavius was isolated in three sputum samples, a diagnosis of chronic cavitary pulmonary aspergillosis was made, and treatment with intravenous amphotericin B was started. An initially clinical improvement was noted, and a first episode of minor hemoptysis was treated with conservative measures. Unfortunately a second major episode of hemoptysis occurred and he died almost immediately. Aspergilloma is defined as the presence of a fungus ball inside a preexisting pulmonary cavity or dilated airway and is one of the clinical conditions associated with the clinical spectrum of pulmonary colonization. Tuberculosis is the most common underling disease. Hemoptysis is the most common symptom. Antifungal antibiotics, surgical interventions, bronchial arteries embolization, and intracavity infusion of antibiotics have been proposed without always adequate sufficiency.

## 1. Introduction

Aspergillus spp are ubiquitous soil-dwelling microorganisms found in dust, foods, organic materials, compost, spices, and rotted plants, and they colonize human respiratory system primary through airborne spore's inhalation. Although there are almost 200 known species of Aspergillus, only a few provoke human disease. Aspergillus fumigates and aspergillus niger are the most encountered, others like aspergillus flavius, aspergillus clavatus, aspergillus niveus, aspergillus terreus, or aspergillus nidulans are also to be responsible in some cases [1, 2].

## 2. Case Presentation

**Figure 1:**
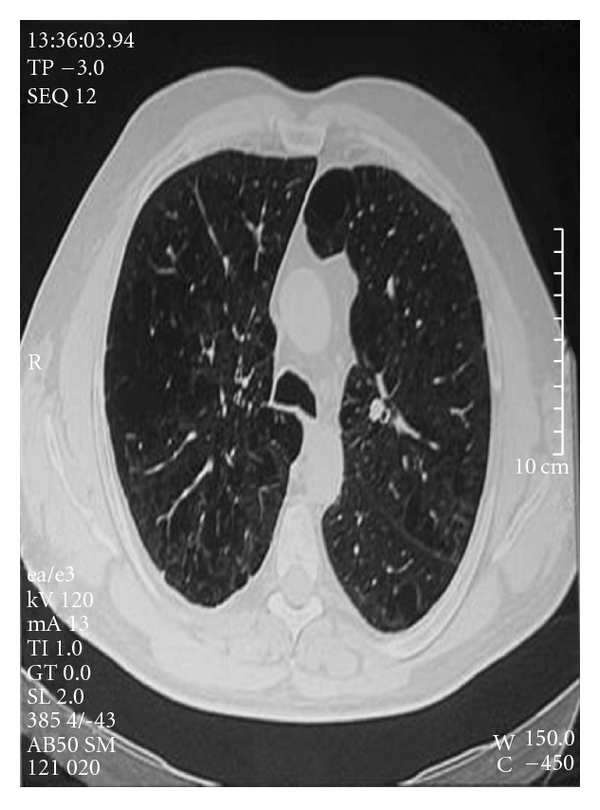
Two years before his admission he was presented with dyspnea on exception, and on CT imaging emphysema was the main evidence.

**Figure 2:**
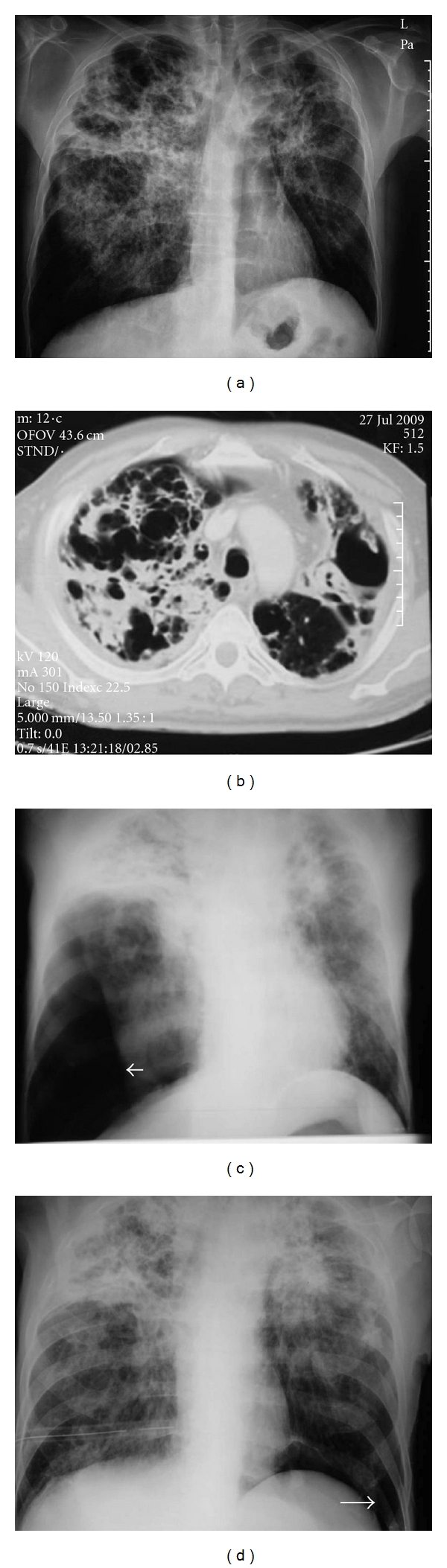
Almost one year prior to his admission he was presented with weight loss, fever, and dyspnea and pulmonary tuberculosis due to mycobacterium avium infection was diagnosed. One month after a bilateral pneumothorax (arrows) was diagnosed and pleurodesis was performed.

**Figure 3:**
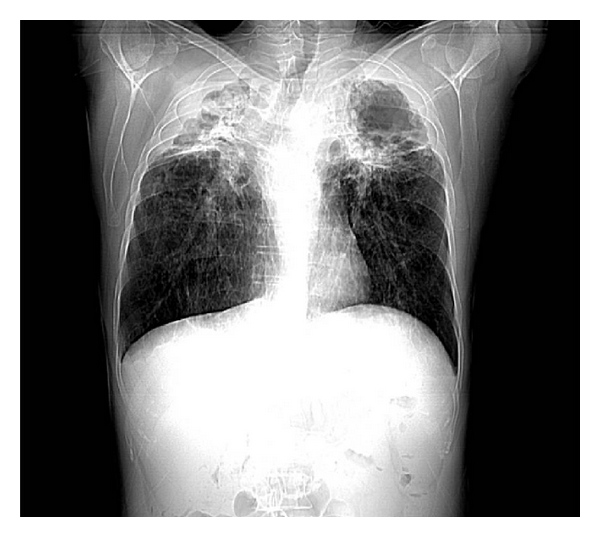
On scanogram a rather catastrophic view is demonstrated including cavitary lesions, extensive fibrosis, and consolidation mainly at the upper lobes.

**Figure 4:**
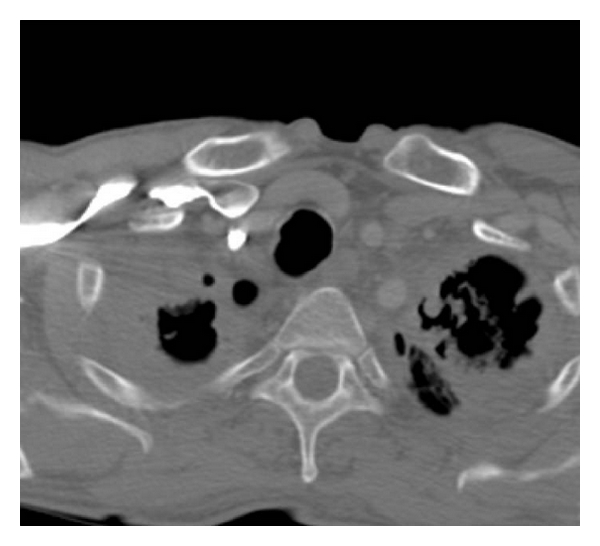
On a CT scan view of the upper lobes cavity lesions are demonstrated, with new shadows inside them, most probably due to aspergillus infection.

A 50-year-old male, smoker (50 p/y), with a history of alcoholism, severe COPD, bilateral pneumothorax, and tuberculosis due to mycobacterium avium infection, was admitted in our department eight months after antituberculosis treatment initialization due to one-month period of high fever, up to 39.6°C, loss of weight, almost 5 kgr, and productive cough with purulent sputum (Figures 1 and 2). Despite adequate treatment with antibiotics, in outpatient setting, he remained with no clinical improvement. Routine laboratory tests on admission revealed increased erythrocyte sedimentation rate (ESR = 134 mm/hr) and C-reactive protein (CRP = 112 mg/dL). A chest X-ray demonstrated cavitations, consolidation, and fibrosis at both upper lobes. Serologic tests for hepatitis A, B, C, D, and human immunodeficiency virus did not reveal any abnormality. Broad spectrum antibiotics were initialized during his hospitalization without any improvement. Finally in three different sputum samples Aspergillus flavius was isolated and, a CT scan demonstrated cavitary lesions with shadows, probably due to fungus contamination inside them, especially in the upper left lobe (Figures 3 and 4). These facts established a diagnosis of chronic cavitary pulmonary aspergillosis. Treatment with amphotericin B provided an initial clinical improvement but eleven days after a first episode of mild hemoptysis occurred and was controlled with conservative measures. Eighteen days after treatment initialization a second episode of hemoptysis occurred and, although he was intubated immediately, his cardiorespiratory system collapsed and he died.

## 3. Discussion

Aspergilloma is defined as the presence of fungus balls inside preexisting cavities or dilated airways and is one form of pulmonary pathology due to the inhabitation of Aspergillus spp into the respiratory system [1]. This fungal ball histology consists of fungal mycelia, fibrin, mucus, inflammatory cells, and tissue debris [4]. A preexisting pulmonary cavity is a key element. Tuberculosis is the most frequent cause of the formation of such condition [1–3, 5, 6]. Sarcoidosis, emphysema, bronchial cysts or bulla, brochiectasis, ankylosing spondylitis, neoplasms, pulmonary infraction, and other infections, even a prior fungal one, have been described to contribute as well [5, 7–10]. 

Recently a prior classification as simple or convex has changed to simple and chronic cavitary pulmonary aspergillosis. Simple aspergillomas are associated with thick-wall cysts and little surrounding parenchymal damage whereas chronic cavitary disease is the more devastating form with thick walls, multiple cavities, and substantial parenchymal changes [2].

Although some aspergillomas remain asymptomatic, and found as an incidental finding on chest radiographs, most became symptomatic. Hemoptysis is the commonest symptom, it occurs to 69% to 83% of all patients, and it ranges from mild to lifethreatening, with a mortality rate ranging from 2% to 14% [6, 11–15]. Other symptoms include fever, malaise, weight loss, productive cough, and clubbing and could be associated with the underlined disease [1–3]. Hemoptysis is believed to be from bronchial origin, but only speculations could be done according to the pathophysiological underling mechanisms. It seems that anastomotic plexus between pulmonary and bronchial arteries around the damaged parenchyma is a main factor to hemoptysis occurrence [4].

Diagnosis of aspergilloma is achieved by a combination of clinical suspicion with radiological, microbiologic, and serologic findings. Radiological imaging of aspergilloma may be difficult in plain radiographs, and chest CT scan is needed to establish diagnosis. The presence of a mobile intracavity mass with an air crescent in the periphery with possible thickening of the adjacent pleura is the main evidence. A change in the position of the fungus ball when the patient is rotated during scan is an interesting finding although in most cases rather variable [16, 17]. Differential diagnosis of such radiographic appearance includes malignancies, abscesses, hydatid cysts, hematomas, and Wegener granulomatosis, and in fact any of these conditions may coexist [18, 19]. Sputum examination may contribute to diagnosis revealing the presence of aspergillus spp although a high possibility of negative examinations, up to 50% in some reports, may be present. Serum IgG antibodies are rather helpful, but in cases of aspergilloma due to species other than Aspergillus fumigatus or in patients under steroid treatment the results could be falsely negative [7].

A nonimmunocompromised patient without any symptoms usually does not require any treatment [2]. Recent guidelines originated from Infectious Diseases Society of America (IDSA) recommend that if the disease progresses or the patient develops hemoptysis treatment with voriconazole (6 mg/kg IV every 12 h for 1 day, followed by 4 mg/kg IV every 12 h, oral dosage is 200 mg every 12 h) or itraconazole could be used [20]. In our case the alternative use of amphotericin B (5 mg/kg/day IV) achieved a substantial improvement without any side effects which always must be in concern. Surgical interventions included cavernostomy, segmentectomy, lobectomy, and pneumonectomy, and they are not considered as first-line therapy due to high morbidity and mortality, although they are improving over time. Postoperative nonfatal complications include prolonged air leak, empyema, incomplete lung expansion, hemorrhage, wound infection, chylothorax, and respiratory insufficiency [21–30]. Due to the above only in selected patients with low surgical risk such surgical invasive procedures combined with antibiotics seem reasonable enough. Bronchial artery embolization is an effective and safe procedure to control hemoptysis, but unfortunately high rates of recurrences and mortality are associated with aspergilloma [31, 32]. Present guidelines recommend embolization as a short-term treatment bridging these procedures to more definite therapy like surgery [20]. Finally some researchers have proposed CT-guided intracavitary infusion of amphotericin B as a complementary therapy in cases of hemoptysis, but the results are still inconclusive [33, 34]. 

In conclusion the inhalation of spores and the colonization of Aspergillus spp into the respiratory system result in a wide spectrum of clinical conditions. The presence of preexisting cavities is the factor that leads to aspergilloma which in cases of severe hemoptysis leads to a rather threatening disease and even death despite adequate treatment. Early clinical suspicion, diagnosis, and multimodality treatment, including antifungal antibiotics, surgery, embolization, or even newer techniques, like intracavitary infusion of antibiotics, are nowadays the treatment options for aspergillomas therapy.
